# N-carbamylglutamate (NCG) supplementation in low-protein diets improving egg quality by regulating protein metabolism pathway of oviduct in laying hens

**DOI:** 10.3389/fcell.2026.1897620

**Published:** 2026-09-03

**Authors:** Mi Wang, Qiulin Liu, Di Han, Jiabo Li, Xing Wang, Wei Ma, Chunqiang Wang

**Affiliations:** 1 College of Animal Husbandry and Veterinary Medicine, Jinzhou Medical University, Jinzhou, China; 2 Collaborative Innovation Center for Prevention and Control of Zoonoses, Jinzhou Medical University, Jinzhou, China; 3 Liaoning Provincial Department of Agriculture and Rural Affairs, Liaoning Provincial Agricultural and Rural Development Service Center, Shenyang, China

**Keywords:** egg quality, laying hens, low-protein diets, NCG, protein metabolism

## Abstract

The objective of this study was to evaluate the effects of low‐protein dietary supplementation with NCG on productivity, egg quality and possible regulatory mechanisms in laying Hens. A total of 150 Beijing Type 2 laying hens at 300 days of age were randomly assigned to five groups: a control group, a low-protein diet group (LP), and three low‐protein diet groups supplemented with 0.06%, 0.12%, or 0.18% NCG. Daily feed intake, Egg number and egg weight were recorded daily on a replicate basis to calculate the egg production rate, average feed intake (ADFI), feed conversion ratio (FCR) and evaluate egg quality. Blood samples were collected from the jugular vein, and the serum was obtained to determine various biochemical parameters using an automatic biochemical analyzer. The protein secretory region of the magnum part of the oviduct was collected and immediately frozen in liquid nitrogen for RNA sequencing analysis. The RNA‐seq results were subsequently validated by real-time quantitative PCR (RT‐qPCR) using Pearson’s correlation analysis. Supplementation with 0.06% and 0.18% NCG in low‐protein diets significantly increased egg weight compared with the LP group (P < 0.05). Albumen height and Haugh unit were significantly increased in the 0.06% NCG group compared with the other groups (P < 0.05). The 0.12% NCG supplementation significantly increased serum BUN content (P < 0.05), whereas the 0.18% NCG supplementation significantly decreased serum phosphorus content (P < 0.05). Serum ALB and NO concentrations were significantly increased in the 0.06% and 0.18% NCG groups compared with the LP group (P < 0.05). Gene Ontology (GO) and Kyoto Encyclopedia of Genes and Genomes (KEGG) pathway analyses were conducted to identify differentially expressed genes and enriched signaling pathways. The results demonstrated that the genes suppressor of cytokine signaling 3 (SOCS3), integrin subunit alpha 8 (ITGA8), platelet‐derived growth factor receptor alpha (PDGFRA), angiotensin‐converting enzyme (ACE), A disintegrin and metalloproteinase with thrombospondin motifs 1 (ADAMTS‐1), and apolipoprotein L domain containing 1 (APOLD1), which are associated with the PI3K/AKT/mTOR signaling pathway, were regulated by NCG supplementation under low‐protein dietary conditions. In conclusion, supplementation with 0.06% NCG in a low-protein diet improved egg weight and egg quality by regulating protein synthesis and secretion in the magnum part of the hen’s oviduct.

## 1 Introduction

Low-protein diets have received increasing attention in the poultry industry due to their significant role in reducing nitrogen emissions (De Cesare et al., 2019; Kobayashi et al., 2013; Macelline et al., 2020). Nutritionists have shown growing interest in reducing dietary protein levels in commercial layer feeds because of the high cost of protein ingredients and increasing environmental pressure to limit nitrogen excretion. Previous studies in broilers (Ospina-Rojas et al., 2014), ducks (Wu et al., 2024), and quails (Wen et al., 2017) have demonstrated that marginal reductions in dietary crude protein content, combined with supplementation of crystalline amino acids, do not adversely affect growth performance while reducing protein intake and nitrogen excretion. Keshavarz and Jackson (KESHAVARZ and JACKSON, 1992) reported that eggs produced by hens fed a low crude protein diet (15% CP) differed in weight from those produced by hens fed a control diet (18% CP).

Hy-Line Brown laying hens consuming reduced-protein diets (15% CP) experienced reductions in laying rate, egg mass, egg weight, and feed efficiency compared to hens on control diets (17% CP) and equivalent energy levels between 33 and 40 weeks of age (Meluzzi et al., 2001). However, reducing dietary protein from 17% to 15.5% resulted in a 14.9% improvement in feed efficiency without compromising egg quality parameters, compared to the standard 17% CP diet at the same energy level (Nawab et al., 2025).

N-carbamylglutamate (NCG) is an activator of endogenous arginine synthesis (Wu et al., 2012). Previous studies have demonstrated that NCG improves the antioxidant capacity of the mother, fetus, and placenta, promotes fetal and placental growth during pregnancy, regulates blood urea levels, enhances feed efficiency, and improves productive performance (Zhang et al., 2016; Mahdi et al., 2021). NCG enhances endogenous arginine synthesis (Wu et al., 2012) and subsequently increases the availability of arginine-family amino acids, including glutamine, glutamate, proline, ornithine, and citrulline. In addition, NCG promotes follicular development by modulating cholesterol metabolism in yak ovaries (Zhou et al., 2021) and by enhancing ovarian angiogenesis in chickens (Zhang et al., 2023). Dietary supplementation with NCG has also been shown to promote follicular development in laying hens (Ma et al., 2020). As an activator of endogenous arginine synthesis, NCG has the potential to regulate laying performance and egg quality by modulating uterine function in laying hens (Ma et al., 2023a).

In the present study, the effects of graded levels of NCG supplementation in low-protein diets on production performance and egg quality in laying hens were first evaluated. Uterine tissue was subsequently collected for transcriptomic analysis using RNA-seq. Therefore, the objective of this study was to investigate the effects of dietary NCG supplementation in low-protein diets on production performance, egg quality, and possible regulatory mechanisms in laying hens, thereby providing mechanistically grounded information to support targeted for improving egg quality in laying hens actual production.

## 2 Materials and methods

### 2.1 Animals, diets, and experimental design

A total of 150 Beijing Type 1 laying hens at 300 days of age (body weight 1.6 ± 0.1 kg) were randomly allocated to five treatment groups, with ten replicates per group and three hens per replicate. The experimental groups included a control group, a low-protein diet group (LP), and three low-protein diet groups supplemented with 0.06%, 0.12%, or 0.18% NCG. The doses of NCG (0.06%, 0.12%, and 0.18%) were selected based on the study by Ma et al. (Ma et al., 2023b). The detailed feed formulations and nutrient levels for each group are presented in Table 1 and were formulated according to the “Jinghong No. 1 Egg Breeding Chicken Rearing Standard”.

**TABLE 1:** Calculated composition of basal diets and nutrient levels.

Composition	CON	LP	Nutrient level	CON	LP
Ingredients					
Con	64.3	69.46	ME (MJ/kg)	11.94	11.96
Soybean meal	24.5	18.5	CP(%)	16.00	14.00
Limestone	8.5	8.5	Calcium (%)	3.47	3.47
Soybean oil	1	0.5	Total phosphorus (%)	0.45	0.44
Dicalcium phosphate	0.7	0.85	Lysine (%)	0.851	0.849
Salt	0.25	0.25	Methionine (%)	0.379	0.376
DL-methionine	0.15	0.17	Methionine + cystine (%)	0.631	0.623
Lysine	0.10	0.36	Threonine	0.612	0.608
Threonine	​	0.09	Tryptophan	0.190	0.188
Tryptophan	​	0.02	Valine	0.759	0.758
Valine	​	0.5	Isoleucine	0.610	0.609
Isoleucine	​	0.1	Arginine	1.130	1.14
Arginine	​	0.2	​	​	​
Premix	0.5	0.5	​	​	​
Total	100	100	​	​	​

^a^ Supplied per kilogram of total diet: Fe, 70 mg; Cu, 20 mg; Zn, 70 mg; Se, 0.5 mg; Mn, 4 mg; vitamin D_3_, 0.1 mg; vitamin E, 13 mg; vitamin K_3_; vitamin B_1_, 0.96 mg; vitamin B_2_, 3.2 mg; vitamin B_6_, 2.1 mg; nicotinic acid, 10.7 mg; folic acid, 1.5 mg; calcium D-pantothenate, 4 mg; vitamin B_12_, 0.04 mg; biotin, 0.4 mg; choline chloride, 400 mg.

^b^ Control group (CON), a low-protein diet group (LP).

The experiment lasted for 7 weeks. The first week served as a pre-feeding period, during which all groups except the control group were fed a low-protein diet for the first 3 days. From the fourth day onward, the treatment groups were switched to their respective experimental diets, and the formal experimental period began in the second week. All hens were fed quantitatively at 120 g per hen per day, divided into two feedings at 07:00 and 15:00, with free access to water. The lighting regimen was maintained at 16 h of light and 8 h of darkness.

### 2.2 Production performance analysis

Feed intake of each replicate (pen) was determined by subtracting the weight of the feed refused from the total feed offered. This practice enabled precise tracking of feed intake patterns and consistency among treatment groups. Hen housed egg production rate, weight of eggs and feed conversion ratio (FCR, g feed/g DEM) were computed. The average feed intake (ADFI) per hen was computed by dividing the total feed intake of each pen with number of hens per pen multiplied by number of experimental days.

### 2.3 Egg quality analysis

On the final day of the experiment, ten eggs were randomly selected from each treatment group for egg quality assessment. Eggshell thickness, excluding the inner membrane, was measured using a Vernier caliper and calculated as the mean thickness of the rounded end, pointed end, and equatorial region of the egg. Eggshell strength was determined accordingly. In addition, albumen height and Haugh unit were measured using an egg multi-tester (Touhoku Rhythm Co., Ltd., Tokyo, Japan).

### 2.4 Serum biochemical parameter analysis

Serum concentrations of total protein (TP), albumin (ALB), globulin (GLB), blood urea nitrogen (BUN), calcium (Ca), total phosphorus (P), nitric oxide (NO), and arginine (Arg) were determined using an automatic biochemical analyzer (BK1200, Biobase, Jinan, Shandong, China). All procedures were conducted in accordance with the manufacturer’s instructions.

### 2.5 Preparation of tissue sections of the magnum part of the hen’s oviduct

All segments of the oviduct, including the infundibulum, magnum, isthmus, shell gland, and vagina, were collected from laying hens at the end of the 7-week experimental period. The tissues were immediately fixed in 10% formalin prepared in PBS for 24 h and subsequently embedded in paraffin. Longitudinal sections of the protein-secretory part of the fallopian tube with a thickness of 6 μm were cut and stained with hematoxylin and eosin on glass slides. After deparaffinization and staining, the tissue sections were sealed with neutral resin and covered with cover glasses. Histological characteristics were examined under a light microscope.

### 2.6 RNA sequencing

A total of 18 samples from the protein-secretory part were sequenced using the HiSeq 2500 platform (Illumina, Inc., San Diego, CA, United States of America). Total RNA was extracted from tissue samples using Trizol reagent. The degree of RNA degradation was analyzed by agarose gel electrophoresis and RNA purity was detected using a Nanodrop 2000 spectrophotometer. RNA purification, reverse transcription, library construction and sequencing were performed according to the manufacturer’s instructions (Illumina, San Diego, CA). The RNA concentration was accurately quantified by Qubit 2.0; and RNA integrity was detected using the Agilent 2100 Bioanalyzer. Following sample testing, a total amount of 3 µg RNA per sample was used as input material for the RNA sample preparations. Sequencing libraries were generated using NEBNext® Ultra™ RNA Library Prep Kit for Illumina R (NEB, United States of America) following manufacturer’s recommendations. The library preparations were sequenced on an Illumina HiSeq 2500 platform. Raw data of Fastq format were initially processed by in-house perl scripts. The clean reads were separately aligned to reference genome with orientation mode using HISAT2 software. For each transcription region, StringTie software was used to calculate an FPKM (fragments per kb per million reads) value to quantify its expression levels and variation. The chicken’s genome sequence (90 version) was downloaded from genome website (ftp://ftp.ensembl.org/pub/current_fasta/gallus_gallus/dna/Gallus_gallus.Gallus_gallus-5.0.dna.toplevel.fa.gz). To identify DEGs (differential expression genes) between two different samples, the expression level of each transcript was calculated according to the transcripts per million reads (TPM) method. Essentially, differential expression analysis was performed using the DESeq2 (http://bioconductor.org/packages/stats/bioc/DESeq2/). DEGs with |log2FC|≧1 and FDR<0.05 were considered to be significantly different expressed genes. Gene ontology (GO) (http://www.geneontology.org/) enrichment analysis of differentially expressed genes was performed using GOseq based on Wallenius non-central hypergeometric distribution. Pathway enrichment was assessed using the KEGG (Kyoto Encyclopedia of Genes and Genomes) database (http://www.genome.jp/kegg/). In addition, functional-enrichment analysis including GO and KEGG was performed to identify which DEGs were significantly enriched in GO terms and metabolic pathways at Bonferroni-corrected P-value<0.05 compared with the whole-transcriptome background. GO functional enrichment and KEGG pathway analysis were carried using Goatools and Python scipy software, respectively.

### 2.7 Real-time quantitative PCR

Based on the optimal production performance, egg quality and Serum biochemical observed in the experiments, the 0.06% NCG group was selected for subsequent RNA-seq analysis to elucidate the underlying molecular mechanisms. To validate the differential expression of genes identified by RNA-seq, real-time quantitative polymerase chain reaction (RT-qPCR) was performed for 10 randomly selected differentially expressed genes (DEGs) using the same RNA samples. Gene expression levels were quantified using an RT-qPCR system (7500 Real-Time PCR System, Singapore). Primer sequences for laying hens (Table 2) were designed using Primer 5.0 to span introns and avoid genomic DNA contamination. Total RNA was extracted using TRIzol reagent, and first-strand cDNA was synthesized using the SuperScript First-Strand Synthesis System (Life Technologies, Grand Island, NY). The concentration of purified cDNA was determined by RT-qPCR. PCR products were verified by 1% agarose gel electrophoresis and DNA sequencing. Standard curves were generated using pooled cDNA samples, and relative mRNA expression levels were calculated using the comparative cycle threshold method (2^−ΔΔCt^).

**TABLE 2:** Parameters of primer pairs for RT-qPCR.

Gene	Primer sequence	Product size	GenBank accession
GPR39	F:5′- CAG​CTC​TTC​CAC​AAG​GAG​CA-3′	133bp	NM_001080105.1
​	R:5GCTCATCTCCAGTGGCACTT-3′	​	​
ACE	F:5′- CGT​GCC​AAG​TAC​CAG​GAG​TT-3′	94bp	NM_001167732.2
​	R:5CATACCAGGAGCGCCAGTAG-3′	​	​
ADAMTS1	F:5′- GGC​TGT​GAC​CGG​ATG​ATA​GG	171bp	XM_015299177.4
​	R:5′- CCG​CTG​CTT​CAC​CTC​GAT​AT	​	​
SOCS3	F:5′- CAG​GAG​AGC​GGC​TTC​TAC​TG-3′	134bp	NM_204600.2
​	R:5′- GTC​TTG​ACG​CTG​AGG​GTG​AA-3′	​	​
ITGA8	F:5′- GGA​CCG​TTG​CAC​TGT​CAA​AC-3′	138bp	NM_205288.3
​	R:5′-GGCTACCTCCCTTCTCCTGA-3′	​	​
PDGFRA	F:5′- TGA​AAA​GCG​ATC​ACC​CAG​CT-3′	160bp	NM_204749.3
​	R:5′-TCAGGCAGGGGGATGATGTA-3′	​	​
APOLD1	F:5′- ACC​TCC​GAT​CTC​TCT​GCT​GT-3′	157bp	XM_004937676.5
​	R:5′- CTG​ACT​GGA​GAA​GCT​TGG​GG-3′	​	​
SDC3	F:5′- GGA​CAC​GTC​TCC​TGA​GCA​AA-3′	132bp	NM_205383.2
​	R:5′- GTG​GTG​GAA​GTG​GTA​CTG​GG-3′	​	​
ADD2	F:5′- TTG​GCT​GAA​GGC​TGA​TGA​GG-3′	99bp	XM_046903445.1
​	R:5′- TCC​TGA​GGG​TCG​GTG​TAG​AG-3′	​	​
WNT7A	F:5′- ATC​TTC​CTG​AGC​CTG​GGG​AT	163bp	NM_204292.3
​	R:5′- ACC​CTT​CCC​CGA​TGA​CGA​TA	​	​
β-actin	F:5′- TGCGTGACATCAAGGAGAAG-3′	183bp	NM_205518.1
​	R:5′- TGCCAGGGTACATTGTGGTA-3′	​	​

### 2.8 Statistical analysis

Experimental data were analyzed using one-way analysis of variance (ANOVA), and Duncan’s multiple range test was used for multiple comparisons. The experiment was a completely randomized design with 5 treatments, and 10 replicates were set for each treatment. Statistical analyses were performed using SPSS software version 20.0 (IBM, 2011). The calculated ΔCt value was obtained by subtracting the mean Ct value of the internal reference gene from that of the target gene, and ΔΔCt was calculated as the ΔCt value minus the mean ΔCt of the control group. Differences among treatment means were considered statistically significant at p < 0.05. All results are presented as mean values ± standard deviation.

## 3 Results

### 3.1 Production performance and egg quality

Compared with the control group, no significant differences in ADFI, FCR, egg production rate were observed in the LP, 0.12% NCG, or 0.18% NCG groups (*P* > 0.05). Dietary supplementation with 0.06% and 0.18% NCG in low-protein diets resulted in significantly higher egg weight compared with the LP group (*P* < 0.05). Compared with the LP group, the eggshell strength increased in the 0.06% and 0.18% NCG groups, but the difference was not significant (*P* > 0.05). In addition, albumen height and Haugh unit were significantly increased in the 0.06% NCG group compared with the other groups (*P* < 0.05) (Table 3).

**TABLE 3:** Effects of dietary supplementation with NCG on production performance and egg quality in laying hens.

Intems	CON	LP	0.06% NCG	0.12% NCG	0.18% NCG
Average feed intake (ADFI, g)	99.92 ± 0.678	99.81 ± 0.744	100.08 ± 0.533	100.04 ± 0.476	100.04 ± 0.618
Feed conversion ratio (FCR)	2.07 ± 0.0449	2.05 ± 0.0336	2.12 ± 0.0147	2.06 ± 0.0477	2.01 ± 0.0205
Egg production rate (%)	83.60 ± 0 .04.58^a^	83.13 ± 2.58^a^	79.44 ± 1.90^b^	83.73 ± 2.20^a^	83.75 ± 2.23^a^
Egg weight (g)	59.06 ± 1.51^ab^	57.74 ± 2.12^bc^	59.11 ± 3.63^a^	58.04 ± 0.998^b^	59.35 ± 3.01^a^
Eggshell thickness (mm)	0.292 ± 0.023^ab^	0.299 ± 0.040^a^	0.266 ± 0.047^b^	0.275 ± 0.035^ab^	0.292 ± 0.032^ab^
Eggshell strength (Pa)	39.422 ± 8.830^a^	31.28 ± 6.2021^bc^	34.7 ± 10.9452^ab^	25.433 ± 5.0392^c^	34.02 ± 5.7971^abd^
Albumen height (mm)	4.287 ± 1.471^a^	3.960 ± 1.47^a^	5.137 ± 2.285^b^	4.820 ± 1.577^b^	3.910 ± 1.378^a^
Haugh unit	59.61 ± 12.365^bc^	60.633 ± 12.5568^bc^	71.77 ± 15.3214^a^	69.42 ± 12.5052^ab^	54.7 ± 11.1665^c^

a,b,c Different superscripts within a column indicate a significant difference (P < 0.05); control group (CON), a low-protein diet group (LP).

### 3.2 Serum biochemistry assays

Compared with the control group, neither the LP nor the NCG-supplemented groups exhibited significant effects on serum TP, GLB, Arg, or Ca^2+^ concentrations (*P* > 0.05). Supplementation with 0.12% NCG significantly increased serum BUN concentration (*P* < 0.05), whereas 0.18% NCG significantly decreased serum phosphorus concentration (*P* < 0.05). The LP group showed significantly increased serum NO and ALB concentrations compared with the control group (*P* < 0.05). Furthermore, supplementation with 0.06% and 0.18% NCG significantly increased serum ALB and NO concentrations compared with the LP group (*P* < 0.05) (Table 4).

**TABLE 4:** Effects of dietary supplementation with NCG on on serum biochemical parameters in laying hens.

​	CON	LP	0.06% NCG	0.12% NCG	0.18% NCG
TP (g/L)	60.167 ± 2.558	54.133 ± 11.482	60.167 ± 2.318	70.633 ± 17.00	60.167 ± 8.053
ALB (g/L)	15.567 ± 1.266^a^	12.900 ± 1.453^b^	16.133 ± 0.907^a^	13.733 ± 0.681^ab^	15.767 ± 2.268^a^
GLB (g/L)	42.267 ± 6.555	41.200 ± 12.834	44.033 ± 2.411	52.600 ± 19.058	44.400 ± 8.839
BUN(mmol/L)	1.867 ± 0.2957^b^	2.283 ± 1.2359^ab^	1.623 ± 0.2914^b^	3.753 ± 1.4911^a^	2.127 ± 0.7497^ab^
NO(μmol/L)	21.310 ± 5.516^a^	14.973 ± 2.183b	26.993 ± 2.830a	22.950 ± 0.330^a^	26.830 ± 2.627^a^
Arg (pmol/L)	144.85 ± 38.3344	126.12 ± 22.7346	128.40 ± 15.9118	145.66 ± 16.8712	137.15 ± 27.9896
Ca^2+^(mmol/L)	4.500 ± 0.200	3.910 ± 1.3547	4.340 ± 0.34044	4.7467 ± 0.32517	4.4233 ± 0.53519
P (mmol/L)	3.5567 ± 0.12583^a^	2.8467 ± 1.14054^ab^	2.9767 ± 0.14742^ab^	3.4400 ± 1.0547^ab^	2.1767 ± 0.64902^b^

a,b,c Different superscripts within a column indicate a significant difference (P < 0.05); control group (CON), a low-protein diet group (LP); total protein (TP), albumin (ALB), globulin (GLB), blood urea nitrogen (BUN), calcium (Ca), total phosphorus (P), nitric oxide (NO), and arginine (Arg).

### 3.3 Effects of NCG on the morphology of magnum of the hen’s oviduct

**FIGURE 1:**
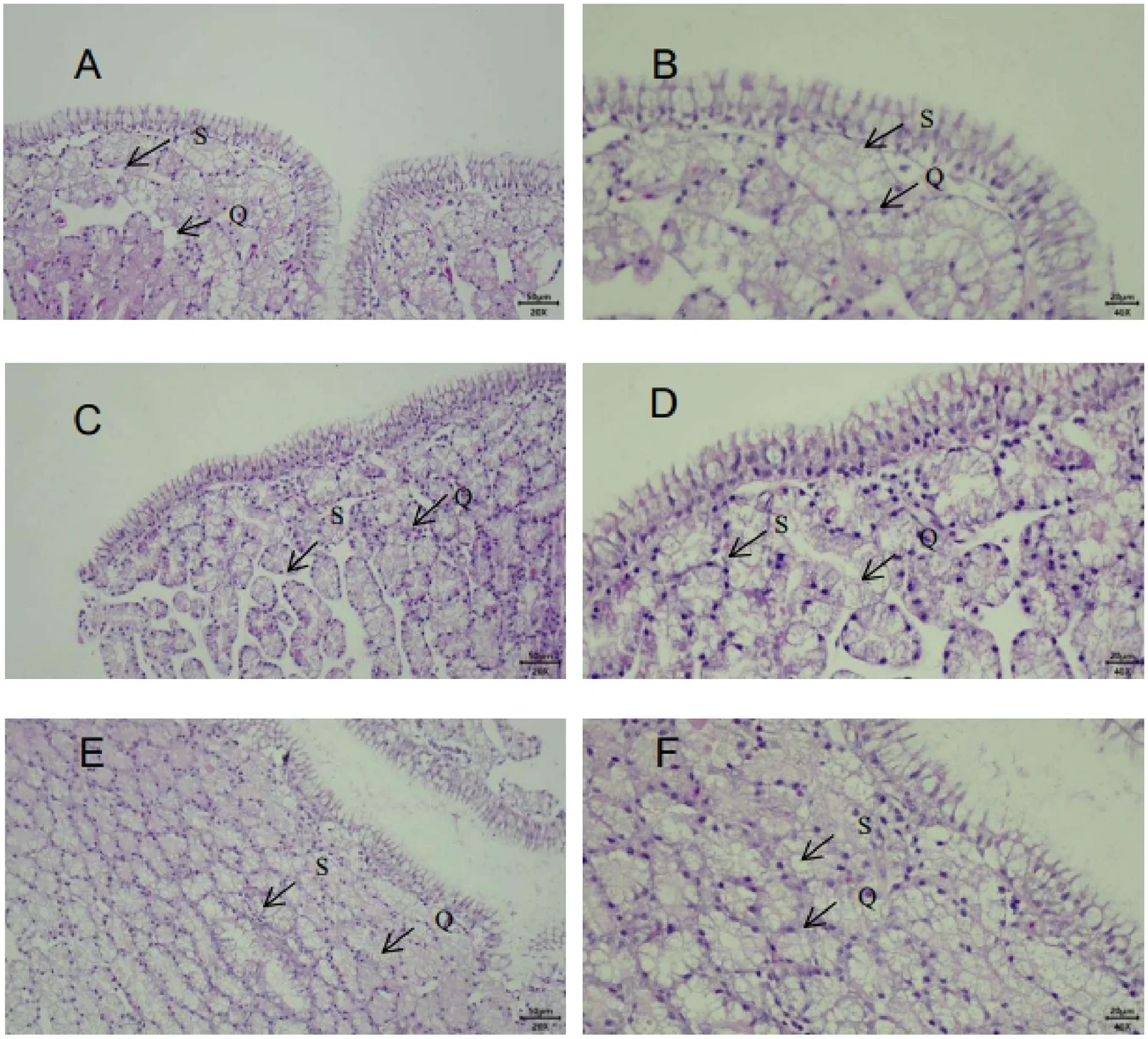
Histomorphology of the magnum part of the hen’s oviduct during the experiment. **(A)**, control (20×); **(B)**, control (40×); **(C)**, low-protein (20×); **(D)**, low-protein (40×); **(E)**, 0.06% NCG (20×); **(F)**, 0.06% NCG (40×). Letters Q and S indicate resting and secretory glands, respectively.

Histological examination of hematoxylin and eosin-stained sections of the protein-secretory region from the control, LP, and 0.06% NCG groups revealed distinct morphological features of the tubular glands (Figure 1). During the secretory phase, the nuclei were condensed and located at the basal region of the cells, the cytoplasm was filled with intensely eosinophilic granules, and the glandular lumen was filled with albumin. During the resting phase, cytoplasmic vacuolation was observed, and the glandular lumen appeared indistinct. In the LP group, the cytoplasm of the cells in the resting phase was more abundant and denser (Figures 1C,D). In contrast, the cytoplasmic morphology of cells in the 0.06% NCG group was similar to that of the control group, and the glandular lumen was rich in albumin (Figures 1E,F).

### 3.4 Effect of NCG on gene expression in the magnum of the hen’s oviduct

Sequencing data from 18 samples were mapped to the reference genome (Gallus, version 5.0). Table 5 presents a summary of the RNA-seq analysis, including the number of mapped reads and gene detection results. The RNA-seq libraries from the 18 samples were sequenced using the Illumina HiSeq 2500 platform, generating a total of 123.76 Gb of raw paired-end reads. As shown in Table 5, after filtering the raw reads, an average of 45,930,947, 47,278,442, and 45,234,585 clean reads were obtained from the control (C), low-protein (L), and 0.06% NCG (N) groups, respectively. The Q20 and Q30 values exceeded 98% and 94%, respectively, in all groups. The GC content was greater than 50% across all samples.

**TABLE 5:** Summary statistics for sequence quality and alignment information of 18 samples from magnum part of the hen’s oviduct.

Sample	Total reads	Clean paired reads	Clean bases(G)	Q20 (%)	Q30 (%)	GC content (%)	Total mapped (%)	Multiple mapped (%)	Uniquely mapped (%)
C_1	46326196	46326196	6.91	98.25	94.52	46.23	88.36	6.05	82.32
C_2	43614090	43614090	6.54	98.26	94.52	47.06	92.74	6.08	86.66
C_3	45322334	45322334	6.77	98.32	94.69	46.41	92.32	5.96	86.36
C_4	46278446	46278446	6.9	98.25	94.53	47.17	94.54	5.89	88.66
C_5	50081740	50081740	7.44	98.22	94.45	47.1	91.35	6.31	85.04
C_6	43962878	43962878	6.52	98.23	94.45	47.64	92.54	6.63	85.91
L_1	42676822	42676822	6.38	98.12	94.23	47.51	92.18	4.13	88.05
L_2	49780940	49780940	7.43	98.15	94.28	47.41	89.22	5.13	84.09
L_3	54621728	54621728	8.14	98.27	94.58	46.34	86.01	6.65	79.37
L_4	41899222	41899222	6.25	97.95	93.75	48.98	88.24	4.49	83.76
L_5	52001872	52001872	7.77	98.29	94.67	47.52	88.19	5.1	83.09
L_6	42690072	42690072	6.34	98.22	94.45	47.32	90.3	5.83	84.48
N_1	42742206	42742206	6.36	98.19	94.36	47.53	90.09	6.12	83.96
N_2	45069654	45069654	6.71	98.21	94.44	47.56	91.94	5.26	86.68
N_3	44895834	44895834	6.66	98.27	94.6	48.77	91.47	5.07	86.4
N_4	44355196	44355196	6.62	98.22	94.46	48.27	92.41	5.83	86.57
N_5	51430704	51430704	7.65	98.3	94.68	47.55	91.36	4.76	86.6
N_6	42913920	42913920	6.39	98.18	94.34	47.41	92.42	5.63	86.78

control group (C), a low-protein diet group (L), 0.06%NCG(N).

**FIGURE 2:**
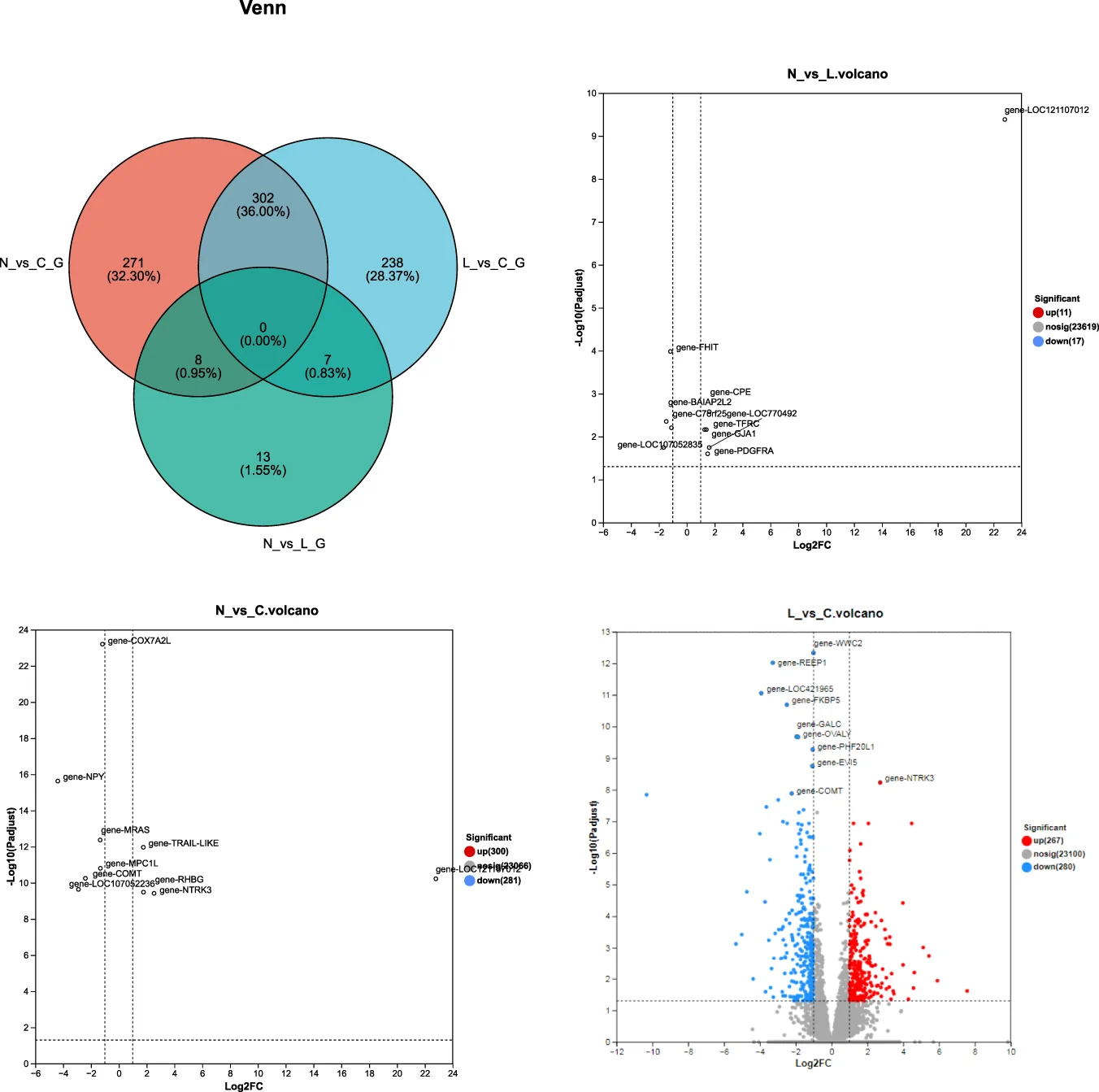
Comparison of differentially expressed genes among the control, low-protein, and 0.06% NCG groups. **(A)** Venn diagram illustrating overlap among functional gene lists. **(B–D)** Scatter plots showing correlations of gene abundance. Red points represent genes upregulated by at least twofold at FDR ≤0.05, blue points represent genes downregulated at the same thresholds, and grey points indicate transcripts with no significant changes.

The Venn diagram (Figure 2A) illustrates the overlap among differentially expressed genes between the NCG/control and NCG/low-protein comparisons, with eight genes shared between these two groups. Seven genes were shared between the low-protein/control and NCG/low-protein comparisons, while 302 genes were shared between the low-protein/control and NCG/control comparisons. Differential expression analysis between control, low-protein, and 0.06% NCG groups was conducted to elucidate gene regulatory patterns. A total of 547 significant DEGs were identified between the control and low-protein groups, including 281 downregulated and 267 upregulated genes. Between the control and 0.06% NCG groups, 581 significant DEGs were detected, comprising 281 downregulated and 300 upregulated genes. Between the low-protein and 0.06% NCG groups, 28 significant DEGs were identified, including 17 downregulated and 11 upregulated genes (Figures 2B–D).

Table 6 lists the top ten significantly upregulated and downregulated genes among the groups based on log2 fold change values. Significantly upregulated genes included G protein-coupled receptor 39 (GPR39), integrin subunit alpha 8 (ITGA8), suppressor of cytokine signaling 3 (SOCS3), apolipoprotein L domain containing 1 (APOLD1), platelet-derived growth factor receptor alpha (PDGFRA), wnt family member 7A (WNT7A), and syndecan 3 (SDC3) in the comparisons of 0.06% NCG/control, low-protein/control, and 0.06% NCG/low-protein. Significantly downregulated genes included APOLD1, angiotensin I converting nzyme (ACE), A disintegrin and metalloproteinase with thrombospondin motifs 1 (ADAMTS-1), WNT7A, and proline-serine-threonine phosphatase interacting protein 1 transcript variant X1 (PSTPIP1) in the same comparisons.

**TABLE 6:** Upregulated and downregulated genes among the 0.06% NCG, control, and low-protein groups.

Gene name	Gene description	Base mean	log2FC	*P*-values	Adjust *P*-values	Up/Down
L Vs. C						
APOLD1	Apolipoprotein L domain containing 1	83.1261	1.247731959	0.002454	0.032774	Down
ACE	Angiotensin I converting enzyme	1472.7404	2.300772895	0.002891	0.036884	Down
ADAMTS1	ADAM metallopeptidase with thrombospondin type 1 motif 1, transcript variant X1	138.1455	0.021285	0.021285	0.021285	Down
WNT7A	Wnt family member 7A	10.6082	−2.18213077	0.000708	0.01336	Down
GPR39	G protein-coupled receptor 39	18.8004	−1.37912774	0.004317	0.047674	Up
N vs. C						
SOCS3	Suppressor of cytokine signaling 3	16.46567	1.45632855	0.000144	0.002994	Up
SDC3	Syndecan 3	10.77937	1.52313874	0.000149	0.003086	Up
ACE	Angiotensin I converting enzyme	1328.5721	−2.8957251	9.77E-08	9.27E-06	Down
PDGFRA	Platelet-derived growth factor receptor, alpha polypeptide	22.5463	1.28937058	0.000207	0.003935	Up
WNT7A	Wnt family member 7A	19.06620	1.97382566	0.001653	0.018291	Up
N vs. L						
APOLD1	Apolipoprotein L domain containing 1	93.50909	1.062371	6.81E-05	0.030257	Up
PSTPIP1	Proline-serine-threonine phosphatase interacting protein 1, transcript variant X1	239.490146	−1.13135	3.48E-05	0.025751	Down
SDC3	Syndecan 3	13.959035	1.501147	6.38E-05	0.030257	UP
PDGFRA	Platelet-derived growth factor receptor, alpha polypeptide	27.723626	1.501549	3.21E-05	0.025127	UP
ITGA8	Integrin subunit alpha 8	86.647509	1.279034	6.08E-05	0.030257	UP

control group (C), a low-protein diet group (L), 0.06%NCG(N).

**FIGURE 3:**
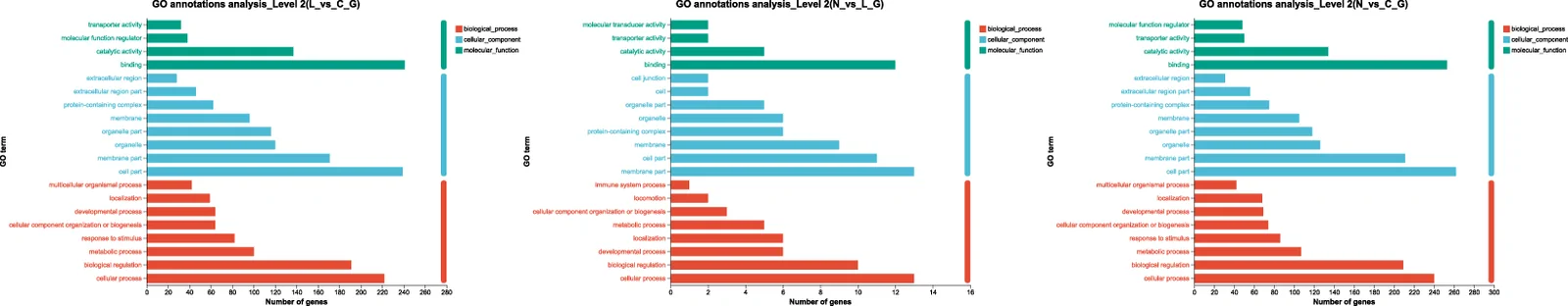
GO analysis of differentially expressed genes in the control, low-protein, and 0.06% NCG groups.

Gene Ontology (GO) is a biological ontology framework consisting of three main categories: biological process, cellular component, and molecular function. Unlike functional annotation of individual genes, GO enrichment analysis evaluates gene sets to reveal the overall functional characteristics of differentially expressed genes. The most significantly enriched GO terms are presented in Figure 3, with positive regulation of metabolic processes representing one of the most prominent enriched terms. Differential GO enrichment in the control/low-protein comparison included signaling receptor binding (*P* < 0.01), cell motility (*P* < 0.01), animal organ development (*P* < 0.01), and extracellular space (*P* < 0.01). Differential GO enrichment in the control/0.06% NCG comparison included transmembrane transporter activity (*P* < 0.01), glycosaminoglycan binding (*P* < 0.01), regulation of transporter activity (*P* < 0.01), and connexin complex (*P* < 0.01). Differential GO enrichment in the 0.06% NCG/low-protein comparison included protein localization to secretory granules (*P* < 0.01), regulation of cell adhesion (*P* < 0.01), lipid transport (*P* < 0.01), plasma membrane protein complex (*P* < 0.01), and growth factor binding (*P* < 0.01) (Table 7).

**TABLE 7:** Important GO enrichment results.

GO ID	Gene functional description	GO functional classification	P-value	Gene number
GO:0005102	Signaling receptor binding	MF	0.00011673846	37
GO:0048870	Cell motility	BP	0.00186372466	21
GO:0048513	Animal organ development	BP	0.00552178107	23
GO:0005615	Extracellular space	CC	0.00000211449	38
GO:0022857	Transmembrane transporter activity	MF	0.0000146175	48
GO:0005539	Glycosaminoglycan binding	MF	0.00003393588	11
GO:0032409	Regulation of transporter activity	BP	0.00015583207	10
GO:0005922	Connexin complex	CC	0.00295456614	4
GO:0033366	Protein localization to secretory granule	BP	0.00276464037	1
GO:0030155	Regulation of cell adhesion	BP	0.00312577174	3
GO:0006869	Lipid transport	BP	0.00071840188	3
GO:0098797	Plasma membrane protein complex	CC	0.00513478461	3
GO:0019838	Growth factor binding	MF	0.00402423152	2

**FIGURE 4:**
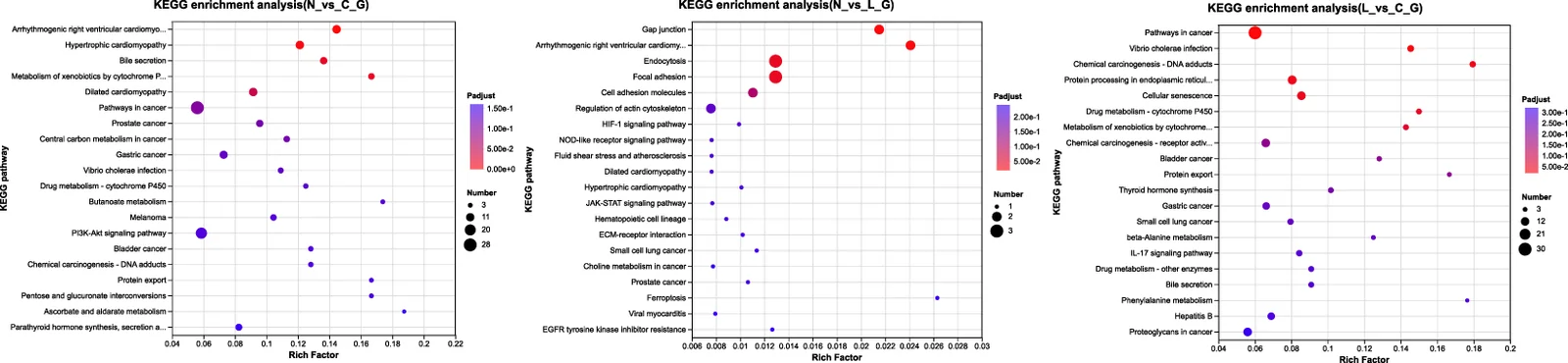
Bubble chart showing significantly enriched pathways identified by Kyoto Encyclopedia of Genes and Genomes (KEGG) pathway analysis based on differentially expressed genes (*P <* 0.05). The x-axis represents the rich factor, defined as the ratio of the number of DEGs enriched in a pathway to the total number of genes in the background gene set. The y-axis represents the enriched pathways. Bubble color indicates enrichment significance, and bubble size represents the number of enriched DEGs.

To further elucidate the major biochemical, metabolic, and signal transduction pathways associated with the DEGs, KEGG pathway enrichment analysis was performed. The results showed that 581 DEGs from the control/0.06% NCG comparison were annotated to 291 pathways, 547 DEGs from the control/low-protein comparison were annotated to 290 pathways, and 28 DEGs from the low-protein/0.06% NCG comparison were annotated to 14 pathways. The most important pathways are shown in Figure 4. Differentially enriched pathways in the control/low-protein comparison included protein processing in the endoplasmic reticulum, cellular senescence, protein export, IL-17 signaling pathway, endocrine resistance, and the Hippo signaling pathway. Differentially enriched pathways in the control/0.06% NCG comparison included hypertrophic cardiomyopathy, protein export, protein processing in the endoplasmic reticulum, PI3K-AKT signaling pathway, Mitogen-activated protein kinase (MAPK) signaling pathway, and ECM-receptor interaction. Differentially enriched pathways in the low-protein/0.06% NCG comparison included gap junction, focal adhesion, cell adhesion molecules, PI3K-AKT signaling pathway, and regulation of the actin cytoskeleton (Table 8).

**TABLE 8:** Important KEGG pathways.

KEGG pathway	Pathway definition	P-value	Gene name
Low-protein/Control			
map04141	Protein processing in endoplasmic reticulum	0.000718452	EDEM1/CAPN2/ATXN3
map04218	Cellular senescence	0.000622707	MTOR/CDKN1A
map03060	Protein export	0.005140759	SEC61B
map04657	IL-17 signaling pathway	0.011787812	MUC5ACL/PTGS2
map01522	Endocrine resistance	0.047398481	MTOR
map04390	Hippo signaling pathway	0.030939843	WNT7A/WNT16
0.06%NCG/Control			
map05410	Hypertrophic cardiomyorathy	9.76E-06	LMNA/ACE/DES
map03060	Protein export	0.006100203	SRP9/HSPA5/SPCS3
map04141	Protein processing in endoplasmic reticulum	0.020965599	ADORA1
map04151	PI3K-akt signaling pathway	0.004398607	MTOR
map04010	MAPK signaling pathway	0.012147244	FGF1/FGF2/PDGFRA
map04512	ECM-receptor interaction	0.0116364271	LAMC2/G1ITGA5/COMP/ITGB6/ITGA9
0.06%NCG/Low-protein			
map04540	Gap junction	0.003685858	GJA1/PDGFRA
map04510	Focal adhesion	0.00138108	CAV1/ITGA8
map04514	Cell adhesion molecules	0.013356182	SDC3
map04151	PI3K-akt signaling pathway	0.047434136667	ITGA8/PDGFRA
map04810	Regulation of actin cytoskeleton	0.027165339	ITGA8/PDGFRA

**FIGURE 5:**
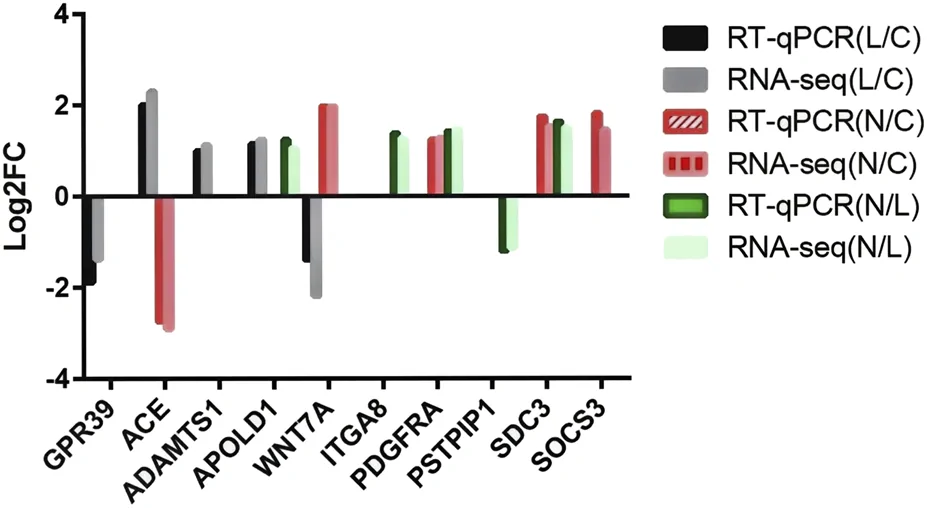
Correlation of mRNA expression levels of ten randomly selected DEGs determined by RNA-seq and RT-qPCR. The x- and y-axes represent log2 ratios measured by RNA-seq and RT-qPCR, respectively. Values are expressed as mean ± SEM (n = 6). Groups include 0.06% NCG, control, and low-protein.

To verify the reliability of the RNA-seq results, ten differentially expressed genes from major enriched pathways were randomly selected, including ten significantly upregulated genes and four significantly downregulated genes. The expression levels of these genes were quantified using RT-qPCR, with β-actin used as the internal reference gene. The results demonstrated that the majority of genes exhibited similar expression patterns between RT-qPCR validation and RNA-seq data, indicating high accuracy and reliability of the transcriptome sequencing results (Figure 5).

## 4 Discussion

### 4.1 Production performance and egg quality analysis

Low-protein diets (CP 14%) have been reported to negatively affect hen productivity, impairing egg weight and egg mass compared with control diets containing 18% crude protein (CP) (Jahan et al., 2023). However, other studies have indicated that daily feed intake and egg production rate did not differ significantly between low-protein (CP 16%) and normal-protein (CP 18%) diets19]. Similarly, Zeweil et al. (Zeweil et al., 2011)reported that daily feed intake was not significantly affected by dietary protein levels of 14% or 16% CP in laying hens during the experimental period of 28–48 weeks of age. In the present study, no significant differences in ADFI, FCR, egg production rate or egg weight were observed between the low-protein group and the control group (*P* > 0.05). Therefore, it is speculated that a 2% reduction in dietary protein level may be within an acceptable range. However, supplementation with NCG in the low-protein diet resulted in significantly increased egg weight compared with the low-protein group (*P* < 0.05). Consistent with these findings, Ma Wei et al. (Ma et al., 2023a) reported that NCG, as an activator of endogenous arginine synthesis, has the potential to regulate laying performance and egg quality by modulating uterine function.

Previous studies by Zeweil et al. (Zeweil et al., 2011)and Alagawany M. et al. (Alagawany et al., 2020)demonstrated that most egg quality traits were not affected by dietary protein levels. In the present study, eggshell strength was significantly decreased in the low-protein group, whereas eggshell thickness, albumen height, and Haugh unit did not show significant changes compared with the control group (*P* > 0.05). Variations in egg quality among studies may be attributed to differences in disease status, stress, environmental temperature, and feeding conditions. Notably, supplementation with 0.06% NCG increased eggshell strength (*P* > 0.05), significantly increased albumen height, and Haugh unit compared with the low-protein group (*P* < 0.05). These findings are consistent with previous research evaluating the effects of NCG on egg quality in laying hens (Ma et al., 2023a).

### 4.2 Serum biochemistry assays

The present results are consistent with the findings of Zhang et al. (Zhang et al., 2021), who reported decreased concentrations of total protein, albumin, and globulin under low feeding levels. In contrast, supplementation with NCG at different levels of concentrate was shown to significantly increase serum albumin concentrations compared with diets without NCG supplementation at a 2% concentrate level (Al-Ani et al., 2022). Albumin concentration is considered an important indicator of liver and kidney function, nutritional status, and the absence of inflammation (Garcovich et al., 2009), reflecting overall physiological health. In the present study, supplementation with 0.06% and 0.18% NCG significantly increased serum ALB concentrations compared with the low-protein diet group (*P* < 0.05).

Consistent with the findings of Sharifi M. R. (Sharifi et al., 2015), the present results demonstrated that the low-protein diet group exhibited significantly decreased serum NO concentrations compared with the control group (*P* < 0.05). NCG is known to enhance endogenous arginine synthesis by activating carbamoyl phosphate synthase 1 and pyrroline-5-carboxylate synthase (Wu et al., 2004). Subsequently, arginine is converted to nitric oxide by nitric oxide synthase, which mediates VEGFA-induced proangiogenic functions. Nitric oxide functions as a vasodilator by relaxing smooth muscle cells within blood vessel walls (Wang et al., 2018). Previous studies have demonstrated that NCG supplementation increases plasma NO concentrations in pigs (Zhu et al., 2015)and lambs (Zhang et al., 2019). Increased NO concentrations promote angiogenesis and enhance nutrient transport capacity. In the present study, supplementation with 0.06% and 0.18% NCG significantly increased serum NO concentrations compared with the low-protein group (*P* < 0.05). These findings explain the observed increase in serum NO levels in NCG-supplemented low-protein groups relative to the low-protein group.

### 4.3 Effect of NCG on gene expression in the magnum part of the hen’s oviduct

The oviduct, particularly the magnum and uterus, plays a critical role in egg formation (Yin et al., 2019). The oviductal magnum is responsible for the synthesis and secretion of egg white proteins; therefore, the health status of the fallopian tube directly affects egg production performance (Madekurozwa and Mpango, 2018). Histological examination of the magnum and tubular glands via Hematoxylin and Eosin (H&E) staining revealed that the cytoplasmic morphology in the 0.06% NCG group was comparable to that of the control group, accompanied by abundant albumen secretion. Although the lack of quantitative morphometric analysis represents a limitation, the observed histological features clearly delineate structural differences among the groups. Based on these findings, we speculate that NCG supplementation in a low-protein diet may modulate protein deposition within the oviduct. However, the precise underlying regulatory mechanism in laying hens remains to be elucidated.

In the present study, RNA sequencing was applied to determine gene expression profiles and metabolic pathways in laying hens fed a low-protein diet supplemented with 0.06% NCG. The RNA-seq data provided high sequencing depth, with 79.09% of reads successfully mapped to the reference genome. The high-quality sequencing data and favorable mapping rates ensured the accuracy and reliability of subsequent differential gene expression analyses. Among the top ten significantly upregulated and downregulated genes identified based on log2 fold change values in the comparisons of 0.06% NCG/control, low-protein/control, and 0.06% NCG/low-protein, the upregulated genes were primarily associated with SOCS3, ITGA8, PDGFRA, and APOLD1, whereas the downregulated genes were mainly associated with APOLD1, ACE, and ADAMTS1. Functional enrichment analyses revealed that the identified GO terms and KEGG pathways were primarily involved in cellular and metabolic processes. To validate the RNA-seq results, several genes were randomly selected for RT-qPCR analysis. A high degree of consistency was observed between RT-qPCR validation and RNA-seq results, which is consistent with findings reported in previous studies in cows (Cui et al., 2014), broilers (Coble et al., 2014), and chickens (Wang et al., 2014).

In the present study, several significant metabolic pathways were identified among the comparisons of 0.06% NCG/control, low-protein/control, and 0.06% NCG/low-protein. These pathways were primarily associated with endocrine resistance (MTOR), hypertrophic cardiomyopathy (ACE), protein processing in the endoplasmic reticulum (APOLD1), PI3K-Akt signaling pathway (MTOR, ITGA8, PDGFRA), MAPK signaling pathway (PDGFRA), gap junction (PDGFRA), focal adhesion (ITGA8), regulation of actin cytoskeleton (ITGA8, PDGFRA), and cell adhesion molecules (SDC3).

APOLD1 is an early response protein expressed in endothelial cells and may play an important role in regulating endothelial signaling pathways and vascular function.

Phosphatidylinositol 3-kinase (PI3K) and serine threonine protein kinase (PKB/AKT), which are regarded as key signal transduction molecules in cells, are associated with a variety of biological processes, including apoptosis, insulin resistance, and adipogenesis (Shaw, 2011; Jiménez-Castro et al., 2013; Pang et al., 2013). APOLD1 has been reported to enhance PI3K/Akt activation (Diaz-Cañestro et al., 2020).

SOCS3 plays an important role in cellular signaling and is involved in regulating physiological processes, inflammation, cell proliferation, and metastasis (Diaz-Cañestro et al., 2020; Haan et al., 2003). The SOCS3 gene is capable of regulating insulin levels by inhibiting signal transduction through the JAK/STAT pathway (Seufert, 2004). Previous studies have identified insulin as the primary ligand for the PI3K/AKT pathway, which regulates lipid and glucose metabolism in essential organs, including the brain, liver, muscle, adipose tissue, and pancreas (Huang et al., 2018). SOCS3 participates in the JAK/STAT signaling pathway, which can interact with other cellular signaling pathways, such as the PI3K/AKT/mTOR pathway.

ADAMTS-1, a member of the ADAM-related protein family, plays a key role in normal growth, organogenesis, and fertility (Sh et al., 2000; Mittaz et al., 2004). ADAMTS-1 is a well-established downstream target of progesterone receptor signaling in the ovary during ovulation (Robker et al., 2009). Previous studies indicate that thrombosis may be prevented by interfering with ADAMTS-1 expression through the PI3K/JNK signaling pathways (Ong et al., 2019).

A study by Mansour S M and Ibrahim R Y M (Mansour and Ibrahim, 2022)indicated that zofenopril was able to suppress the PI3K/Akt signaling cascade either directly by inhibiting its signaling trajectory or indirectly by inhibiting ACE, resulting in reduced angiotensin II levels and decreased cytokines and growth factors, such as VEGF. Vascular reactivity studies using rat aortic rings demonstrated the involvement of nitric oxide synthase, the calcium-calmodulin complex, and the PI3K/Akt pathway in vasodilatory effects (Moreira et al., 2019) Meanwhile, Kim L. reported that ACE could inhibit the PI3K/AKT pathway (Kim et al., 2023).

The results of the present study revealed that NCG supplementation promoted the synthesis of SOCS3 and APOLD1 in ovaries by upregulating their gene expression. A low-protein diet downregulated the mRNA expression of ACE, ADAMTS-1, and APOLD1, which are involved in inhibiting the PI3K/AKT/mTOR signaling pathway.

In this context, these growth-related factors were found to influence cell division and MAPK signaling pathways, particularly the PI3K/AKT/mTOR and Rap1/Ras/MAPK pathways, which are the main pathways associated with cell proliferation and survival (Nakada et al., 2011). In the present study, significant metabolic pathways were detected among the 0.06% NCG/control, low-protein/control, and 0.06% NCG/low-protein comparisons, with most pathways involved in the PI3K-Akt signaling pathway (MTOR, ITGA8, PDGFRA) and the MAPK signaling pathway (PDGFRA).

The PI3K/AKT/mTOR signaling pathway is widely recognized as a central regulatory axis for cell growth, proliferation, protein metabolism, apoptosis, and secretion (Cai et al., 2015). MTOR integrates various extracellular and intracellular signals as part of two protein complexes, mTORC1 and mTORC2. Nitric oxide mediates increases in blood flow and skeletal muscle microvascular perfusion and can also function as a nutrient-signaling molecule capable of activating mTORC1 signaling. As a precursor of arginine, NCG has been shown to activate the mTOR signaling pathway in numerous studies (Zh et al., 2019; Huang et al., 2019; Zeng et al., 2012). MAPK signaling, which includes multiple kinase cascades such as PI3K, JNK, and p38, is widely involved in various cellular functions (Hsueh and Sheng, 1999). Downregulation of PDGFRA has been shown to inhibit STAT3 as well as phosphorylated PI3K (Lin and YongJie, 2020). Comprehensive analyses of ITGA8 revealed its interaction with focal adhesion kinase, leading to activation of the PI3K/AKT signaling pathway (Liu et al., 2025).

In summary, a low-protein diet downregulated the mRNA expression of ACE, ADAMTS-1, and APOLD1, thereby inhibiting the PI3K/AKT/mTOR signaling pathway and affecting protein synthesis and secretion. NCG supplementation in low-protein diets may regulate protein deposition by upregulating the mRNA expression of SOCS3, APOLD1, ITGA8, and PDGFRA, thereby activating the PI3K/AKT/mTOR signaling pathway.

## 5 Conclusion

**FIGURE 6:**
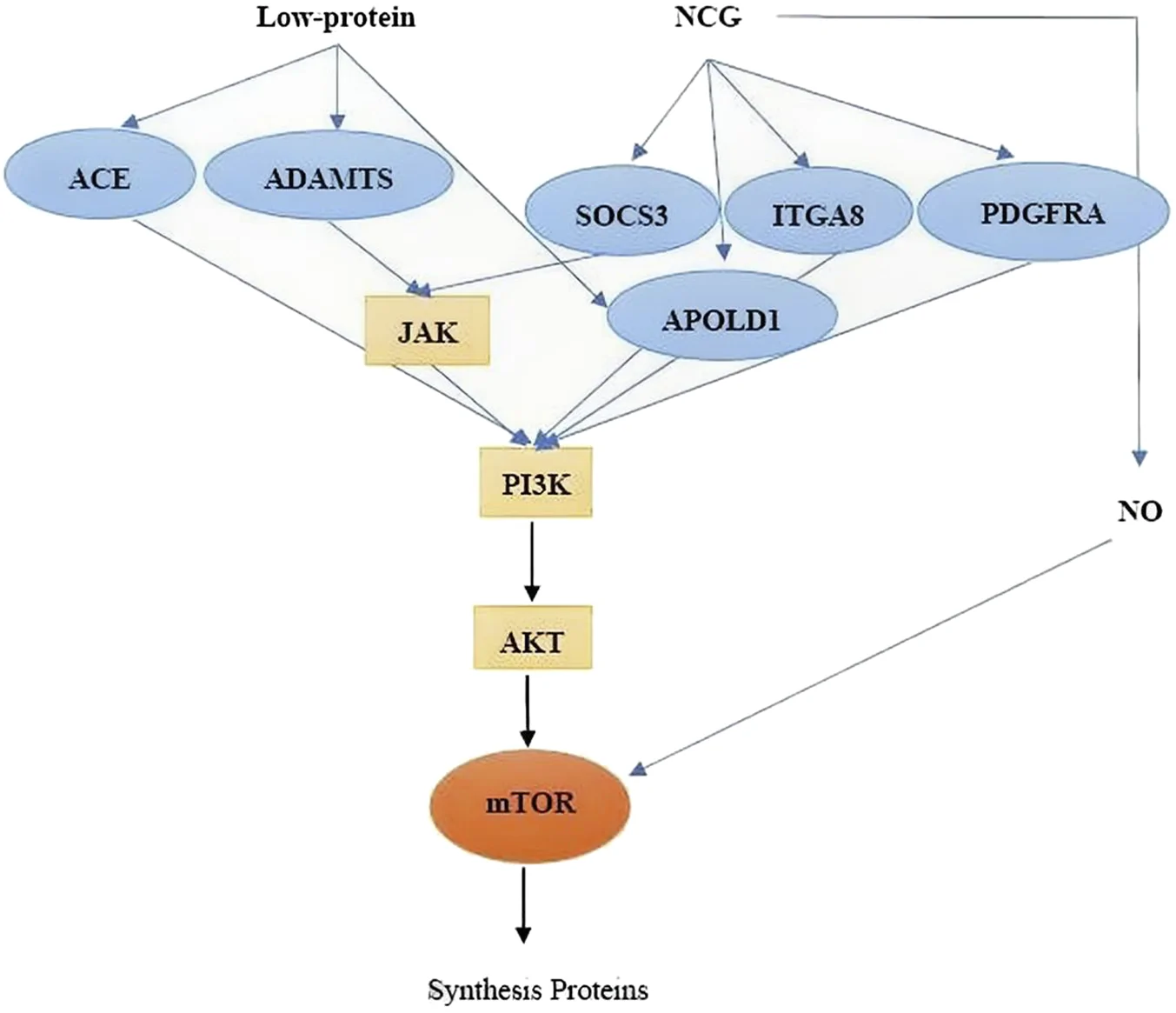
Proposed model of PI3K/AKT/mTOR regulation of gene expression in the magnum part of the hen’s oviduct fed NCG under low-protein conditions.

In conclusion, SOCS3, ITGA8, PDGFRA, ACE, ADAMTS-1, and APOLD1, which are genes associated with the PI3K/AKT/mTOR signaling pathway, were regulated by NCG supplementation and a low-protein diet. The results demonstrated that supplementation with 0.06% NCG in a low-protein diet improved egg weight and egg quality by regulating protein synthesis and secretion in the magnum part of the hen’s oviduct (Figure 6).

## Data Availability

The datasets presented in this study can be found in online repositories. The names of the repository/repositories and accession number(s) can be found below: https://figshare.com/, https://doi.org/10.6084/m9.figshare.32030649.
